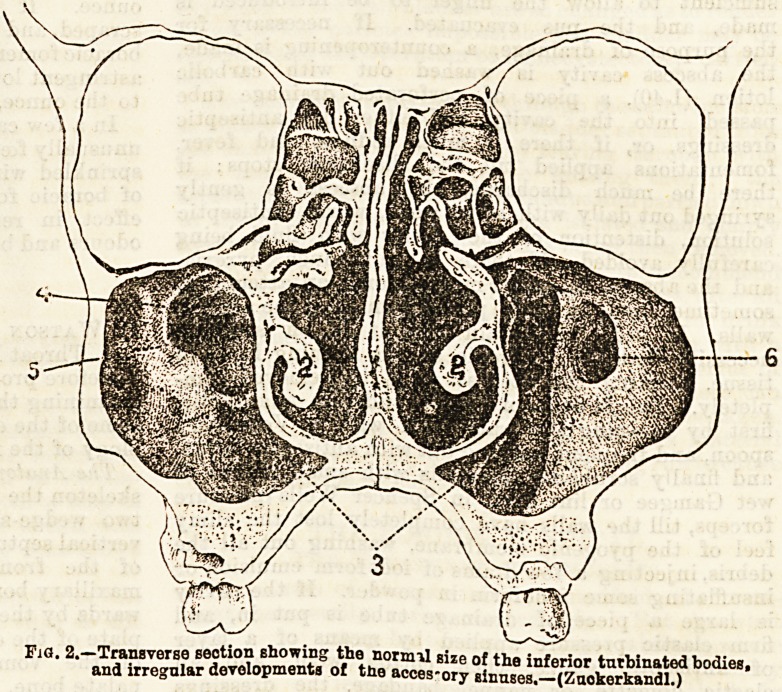# Rhinoscopy

**Published:** 1893-05-13

**Authors:** P. Watson Williams

**Affiliations:** Physician to the Throat Department Bristol Royal Infirmary


### RHINOSCOPY.

P. WATSON WILLIAMS, M.D.Lond., Physician to the Throat Department Bristol Royal Infirmary.

Before proceeding to discuss the clinical methods of examining the nose we propose to very briefly refer to some of the essential points in the anatomy and physiology of the nose and naso-pharynx.

*The Anatomy of the Nose and Naso-pharynx.*—In the skeleton the nasal fossæ or anterior nasal cavities are two wedge-shaped passages separated by a median vertical septum. They communicate with the sinuses of the frontal, ethmoid, sphenoid, and superior maxillary bones. The roof is formed from before backwards by the frontal and nasal bones, the cribriform plate of the ethmoid, the body of the sphenoid, the alæ of the vomer, and the sphenoidal process of the palate bone.

The floor is formed by the palatal plates of the superior maxillary and palate bones and the inner wall of each fossa; the septum by the perpendicular plate of the ethmoid above and the vomer below.

FIG 1.—Section of skull, showing the outer wall of the left nasal fossa 1, the superior, 2, middle, and 3. inferior turbinated bones; 7, dotted outline showing the position of the hiatus semilunaris, and 8. of the ostium, maxillare beneath the middle turbinated bone: 9, a bristle in the nasal duct.; 10, a bristle passed into the infundibulum; 11, bristle, passed through the opening of the sphenoidal cells.

The outer wall, unlike the roof, floor, and septum, is very irregular, owing to the attachment of the turbinated bones. The superior maxillary, nasal, lacrymal, ethmoid, and palate bones take part in its formation.

The superior and inferior turbinated processes of the ethmoid project inwards, forming the superior and middle turbinated bones, and below them is the inferior spongy bone, each being a thin scroll-shaped lamella of bone much curled upon itself. The superior and middle turbinated bones are united anteriorly and diverge posteriorly.

The three turbinated bones divide the outer wall of each fossa forming three passages, the superior, middle, and inferior meatuses.

Beneath the superior turbinated bone are seen posteriorly the openings of the posterior ethmoidal cells and the sphenoidal sinus.

Beneath the middle turbinated bone lies the cruciform process of the ethmoid forming the lower boundary of the crescentic hiatus semilunaris; the superior boundary of this opening being formed anteriorly by the bulla ethmoidalis. At the anterior extremity of the hiatus is the infundibulum or opening by which the frontal sinus and anterior ethmoidal cells communicate with the inferior meatus, and behind this is the ostium maxillare leading to the antrum. Into the inferior meatus opens the lachrymal duct.

These sinuses are not developed till after puberty, with the exception of the maxillary sinus, which is formed during fœtal life.

The roof of the naso-pharynx is formed by the body of the sphenoid and basilar process of the occipital bones, the posterior wall by the body of the atlas, and the lateral walls by the pterygoid plates of the sphenoid.

In the recent state the whole of the fossæ are covered by mucous membrane, which is continued into and lines the accessory sinuses.

In the vestibule, the anterior portion of the nasal fossa roofed in by the nasal cartilages, the mucous membrane is lined with stratified epithelium, and contains several stiff hairs, whose function it is to arrest impurities in the inspired air. In the lower or respiratory portion of the fossæ, *i.e*, up to the middle of the turbinated body and the lower two-thirds of the septum the membrane is lined with ciliated epithelium, is thin, and contains numerous follicular and racemose glands and lymphoid tissue, which here and there is collected into nodules. In the olfactory region—that is to say, in the portions of the fossæ above the respiratory region—the mucous membrane is non-ciliated and columnar. Here the membrane is thicker and softer, highly sensitive, and contains fewer racemose glands, but more of the simpler Bowman's glands. It has a distinctly yellowish colour, and contains the olfactory cells of Max Schultze.

The thickened tissue covering the inferior and middle turbinated bodies has been carefully investigated, more especially by Zuckerkandl. It consists of connective tissue, the superficial surface being covered by ciliated epithelium and the deep portion forming the periosteum. Between the two layers are lymph tissue and an abundant supply of lymphatics, and numerous venous plexuses, into which the capillaries open. Around the venous plexuses unstriped muscular tissue is distributed. The plexuses by distension cause the bodies to swell considerably, and by most rhinologists the vascular tissues of the inferior and middle turbinated bodies are described as erectile tissue. Numerous elastic fibres in the deeper layers cause the tissue to collapse unless actively distended by the peculiarly arranged vascular supply.

Similar erectile tissue is found occupying the lower part of the septum and the floor of the nasal fossæ.

The function of the nose and naso-pharynx in phonation is well known, and the effect of nasal obstruction in modifying the voice in speech and song hardly requires mention. But we must bear in mind that the proper action of the soft palate is essential to clear speech, and that if any condition interferes with its freedom of movement the voice will be impaired. Thus, paralysis of the velum palati, or perforation preventing closure of the naso-pharynx, render it impossible to pronounce the lingual consonants.

The naso-pharynx is continuous with the anterior nasal cavities, and extends downwards as far as the isthmus, the narrow space between draws horizontally

FIG. 2.—Transverse section showing the normal size of the inferior turbinated bodies, and irregular developments of the accessory sinuses.—(Zuckerkandl.)

from the posterior margins of the soft palate and the posterior pharyngeal wall. Into it open the Eustachian tubes by their trumpet-shaped orifices, from the posterior margin of which may be seen the salpingo-pharyngeal folds extending downwards, and forming on each side a fossa between them and the posterior wall of the pharynx, the fossa of Rosenmuller.

The mucous membrane is covered with ciliated columnar epithelium, and is more abundantly supplied with mucous glands than the anterior nasal cavities. Numerous lymphoid follicles exist throughout the pharynx, and a collection of these in the roof and posterior wall of the naso-pharynx forms a glandular mass, similar to the faucial tonsils, and called Luscha's tonsil.

## Figures and Tables

**Fig 1. f1:**
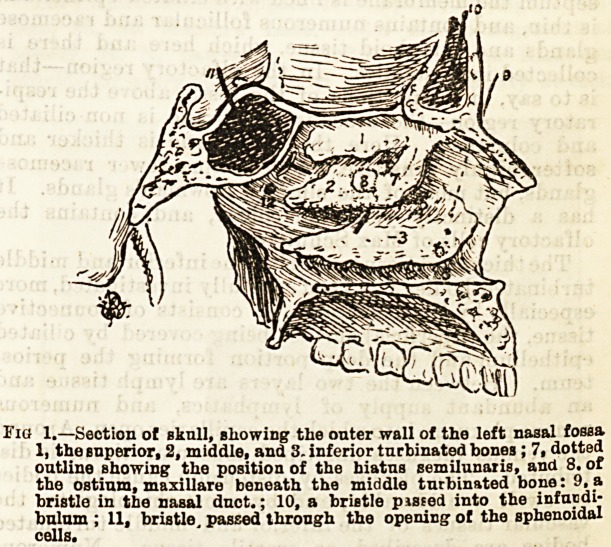


**Fig. 2. f2:**